# FRZB regulates the osteogenic differentiation of periodontal ligament stem cells in an inflammatory microenvironment through Wnt5a-mitochondrial axis

**DOI:** 10.1186/s13619-026-00283-z

**Published:** 2026-02-06

**Authors:** Yuanmeng Su, Houpeng Wang, Tao Luo, Junyao Liu, Xiaoping Hu

**Affiliations:** 1 The Affiliated Stomatological Hospital, Jiangxi Medical College, Nanchang University, Nanchang, 330031 China; 2 Jiangxi Provincial Key Laboratory of Oral Diseases, Nanchang, 330031 China; 3 Jiangxi Provincial Clinical Research Center for Oral Diseases, Nanchang, 330031 China; 4 Third Community Health Service Center, Luodian Town, Baoshan District, Shanghai, Shanghai, 201900 China; 5 School of Basic Medical Sciences, Institute of Biomedical Innovation, Jiangxi Medical College, Nanchang University, Nanchang, 330031 China

**Keywords:** Periodontal Ligament Stem Cells, Frizzled-Related Protein (FRZB), Wnt Signaling Pathway, Mitochondrial Function, Osteogenic Differentiation

## Abstract

The Wnt signaling pathway critically regulates the osteogenic differentiation in periodontal ligament stem cells (PDLSCs). However, the functional contributions of this pathway under inflammatory conditions remain unclear. This study investigated the effect and underlying mechanisms of the FRZB–Wnt5a–mitochondrial axis on the osteogenic differentiation capacity of PDLSCs under inflammatory conditions. PDLSCs were isolated from healthy teeth and exposed to lipopolysaccharide (LPS) to mimic an inflammatory microenvironment. The Wnt pathway-related molecules were assessed, and the osteogenic differentiation capacity and mitochondrial function of PDLSCs were evaluated. To elucidate its regulatory role, we employed gene transfection to establish an FRZB (Frizzled-Related Protein) overexpression model. Results showed that inflammation significantly impaired osteogenic differentiation and activated Wnt/β-catenin signaling. Mitochondrial dysfunction was also observed, including reduced membrane potential, increased calcium and reactive oxygen species (ROS) levels, suppressed autophagic flux, and altered mitochondrial morphology. Notably, FRZB overexpression partially restored mitochondrial function and the osteogenic differentiation capacity of PDLSCs. These results demonstrated that FRZB serves as a pivotal regulator of osteogenic differentiation in PDLSCs. We found that inflammation downregulates FRZB expression, thereby activating Wnt/β-catenin signaling, which leads to mitochondrial dysfunction and ultimately impairs osteogenesis. These findings reveal a mechanism by which inflammation suppresses osteogenesis in PDLSCs and highlight FRZB as a promising therapeutic target for periodontitis.

**Supplementary Information:**

The online version contains supplementary material available at 10.1186/s13619-026-00283-z.

## Background

Periodontitis is recognized as a prevalent chronic inflammatory disease, primarily characterized by periodontal tissue destruction and alveolar bone resorption. It has a high incidence rate, and it is the leading cause of tooth loss in adults, posing a significant threat to human health (Sayad et al. 2020). Periodontal ligament stem cells (PDLSCs) are multipotent stem cells in periodontal tissue with self-renewal and multidirectional differentiation potential. These cells can differentiate into various cell types, including osteoblasts (Liu et al. 2020), fibroblasts (Wen et al. 2025), and cementoblasts (Zhang et al. 2025), etc. They are the most commonly used seed cells in periodontal tissue engineering (Han et al. 2013). In vivo studies have demonstrated that PDLSCs are capable of regenerating alveolar bone, periodontal ligament, and osteoid tissue to repair periodontal defects and are widely used to treat alveolar bone defects caused by periodontal disease (Liu et al. 2025). However, the inflammatory microenvironment impairs the osteogenic differentiation potential of PDLSCs through multiple mechanisms, including alterations in cell signaling pathways and metabolic states (Ru et al. 2023; Zhao et al. 2023a, b; Li et al. 2025).

Mitochondria serve as the primary sites for adenosine triphosphate (ATP) production within cells and are also the main source of reactive oxygen species (ROS), thereby playing a pivotal role in maintaining normal energy, oxidative, and metabolic states (Zhao et al. 2023a, b). Mitochondrial dysfunction is recognized as a critical element in the pathological mechanisms underlying periodontitis (Deng et al. 2024). It encompasses structural and functional impairments of mitochondria induced by various factors, including mitochondrial DNA damage, oxidative stress, dysregulation of mitochondrial dynamics, as well as deficiencies in autophagy and mitophagy. These alterations compromise the ability of mitochondria to execute their normal physiological roles (Jiang et al. 2023). Studies have demonstrated that within the periodontitis microenvironment, PDLSCs exhibit marked mitochondrial dysfunction, manifested by diminished ATP synthesis, reduced mitochondrial membrane potential, and elevated levels of ROS, which collectively contribute to the impairment of their regenerative potential (Li et al. 2022).

FRZB (Frizzled-Related Protein, also known as sFRP3) is a member of the secreted Frizzled-related protein (sFRP) family and acts as an antagonist of Wnt signaling. It regulates the growth and differentiation of specific cell types by directly interacting with Wnt ligands (Yagi et al. 2022). The Wnt signaling pathway is critically involved in regulating the osteogenic differentiation of stem cells (Kawano 2003). Wnt5a, a member of the Wnt family, primarily activates the non-canonical Wnt pathway (Kumawat et al. 2016). However, depending on the receptor context, Wnt5a can also activate the canonical β-catenin pathway, thereby regulating embryonic development and cell fate (Hiong et al. 2015). Notably, within an inflammatory milieu, Wnt5a expression is upregulated, which correlates with a reduction in the osteogenic differentiation potential of PDLSCs (Liu et al. 2024). Inflammatory cytokines, such as TNF-α and IL-1β, can modulate the Wnt signaling pathway through diverse mechanisms, ultimately impairing the osteogenic differentiation capacity of PDLSCs (Su et al. 2025).

Studies have revealed that under chronic inflammatory conditions, activation of the Wnt/β-catenin signaling pathway suppresses mitophagy, leading to the accumulation of damaged mitochondria in mesenchymal stem cells and consequently impairing their osteogenic potential (Zhai et al. 2021). Other research has found that oxidative stress in the inflammatory response can activate the non-canonical Wnt/Ca^2+^ signaling pathway, with excessive Ca^2+^ partially transferring from the endoplasmic reticulum to mitochondria, causing mitochondrial calcium overload and damage (Fei et al. 2025). As a secreted antagonist of Wnt signaling, FRZB acts as a critical negative regulator by specifically binding to Wnt5a and preventing its interaction with cognate receptors, thus attenuating downstream signal propagation (Thysen et al. 2016). Nevertheless, the specific role of FRZB in modulating the osteogenic differentiation of PDLSCs within an inflammatory microenvironment remains inadequately characterized.

The study proposes that the inflammatory microenvironment impairs osteogenic differentiation of PDLSCs through downregulation of FRZB expression, subsequent activation of the Wnt/β-catenin signaling pathway, and resultant mitochondrial dysfunction.

## Results

### Culture of PDLSCs and establishment of periodontitis cell model

PDLSCs migrated from the margins of tissue explants, and their cell density showed a trend of gradually increasing over time. Most cells displayed a homogeneous bipolar spindle-like morphology, characterized by a central bulge, and were organized in whorl-like patterns (Fig. S1A). Flow cytometry analysis revealed that PDLSCs exhibited high positivity for mesenchymal markers CD90 and CD146 (expression ≥ 95%), while showing minimal expression of the hematopoietic marker CD45 (≤ 2%), consistent with a mesenchymal lineage phenotype (Fig. S1B). CCK-8 assays indicated that LPS treatment significantly impaired cell viability in a dose-dependent manner (Fig. S1C). Furthermore, following 24 h of LPS stimulation, the expression levels of inflammatory cytokines (IL-1β, TNF-α, and IL-6) were markedly upregulated, exhibiting a dose-dependent increase that reached a peak at 10 μg/mL. These results confirm that LPS effectively induced an inflammatory response, thereby establishing a reliable in vitro model of periodontitis (Fig. S1D).

### LPS-treated PDLSCs exhibit osteogenic inhibition

Following 21 days of osteogenic induction, Alizarin Red staining revealed distinct mineralization patterns across the experimental groups. No staining was observed in the α-MEM group, whereas the Control group exhibited extensive deposition of intensely stained mineralized nodules. In contrast, the LPS group showed markedly diminished nodule formation, with fewer and lighter-stained nodules compared to the Control group (Fig. S2A). Consistent with these observations, the optical density (OD) value in the LPS group was significantly lower than that in the Control group (Fig. S2B). Collectively, these results indicate that LPS significantly impairs the osteogenic differentiation capacity of PDLSCs.

### LPS-induced inflammation affects FRZB-Wnt5a signaling axis in PDLSCs

**Fig. 1:**
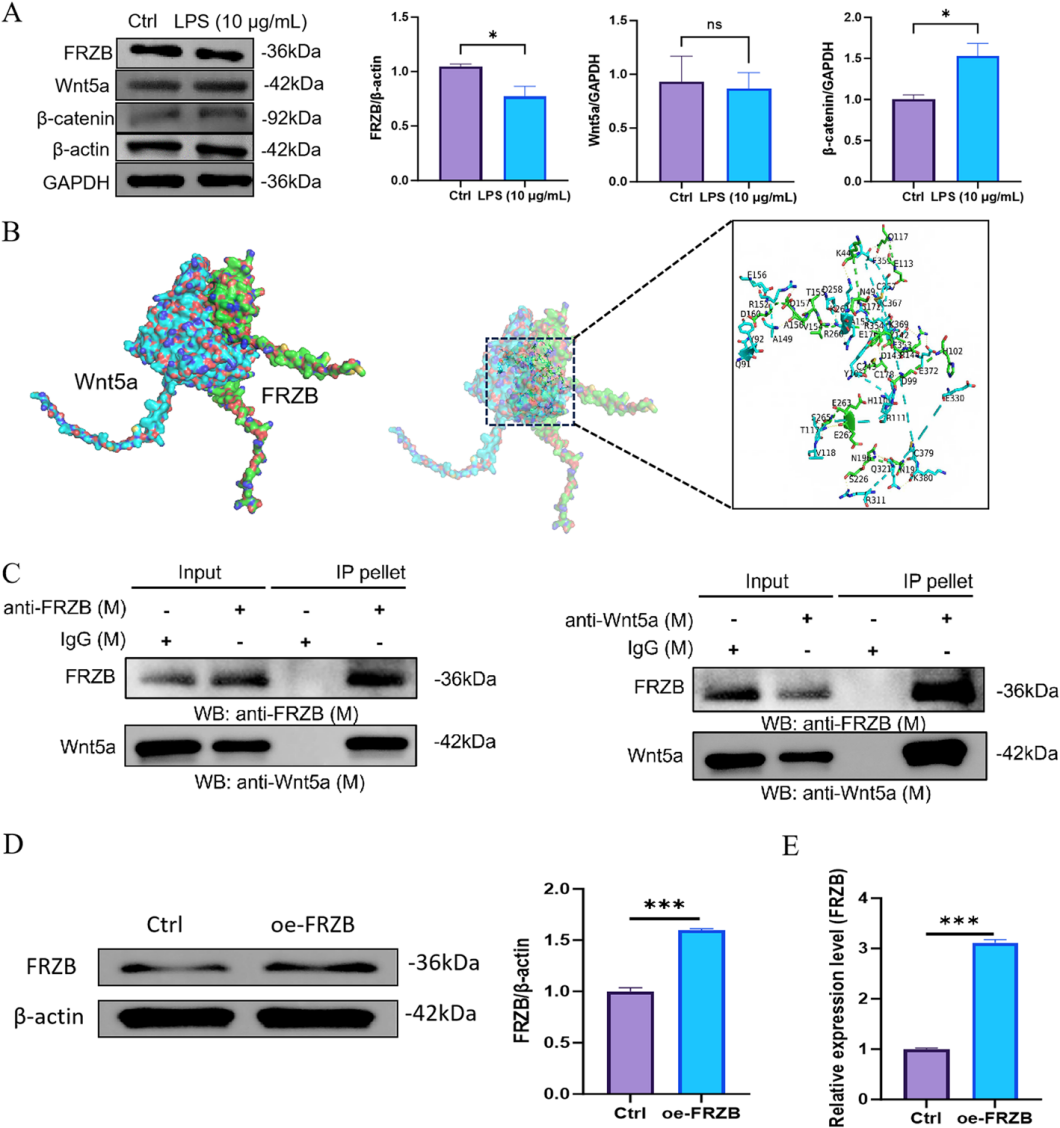
LPS-induced inflammation affects FRZB-Wnt5a pathway in PDLSCs. **A** Western blot was used to examine the protein expression levels of FRZB and Wnt5a in the Control and LPS-treated group, followed by quantitative analysis of the relative expression levels of FRZB, Wnt5a, and β-catenin proteins (data presented as mean ± SD; Standard *t* test; *n* = 3; *P < 0.05). **B** Molecular docking revealed the binding sites between FRZB and Wnt5a. **C** Co-immunoprecipitation confirmed an interaction between FRZB and Wnt5a (*n* = 3). **D** Western blot was used to detect the expression level of FRZB protein in the Control and oe-FRZB group. **E**. qPCR was employed to detect the relative expression level of FRZB (data presented as mean ± SD; Standard *t* test; *n* = 3; ***P < 0.001)

Western blot analysis showed that LPS treatment significantly downregulated FRZB protein expression and markedly increased β-catenin levels, whereas Wnt5a expression remained unaltered (Fig. 1A). Molecular docking further predicted the specific amino acid residues mediating binding at the FRZB–Wnt5a interface (Fig. 1B). Co-immunoprecipitation (Co-IP) confirmed a direct interaction between FRZB and Wnt5a (Fig. 1C). Western blot and qPCR analyses confirmed that FRZB levels were effectively elevated in the oe-FRZB group compared to the Control group, demonstrating the successful establishment of FRZB overexpression (Fig. 1D).

### LPS causes mitochondrial abnormalities, and FRZB overexpression can restore these abnormalities in PDLSCs

**Fig. 2:**
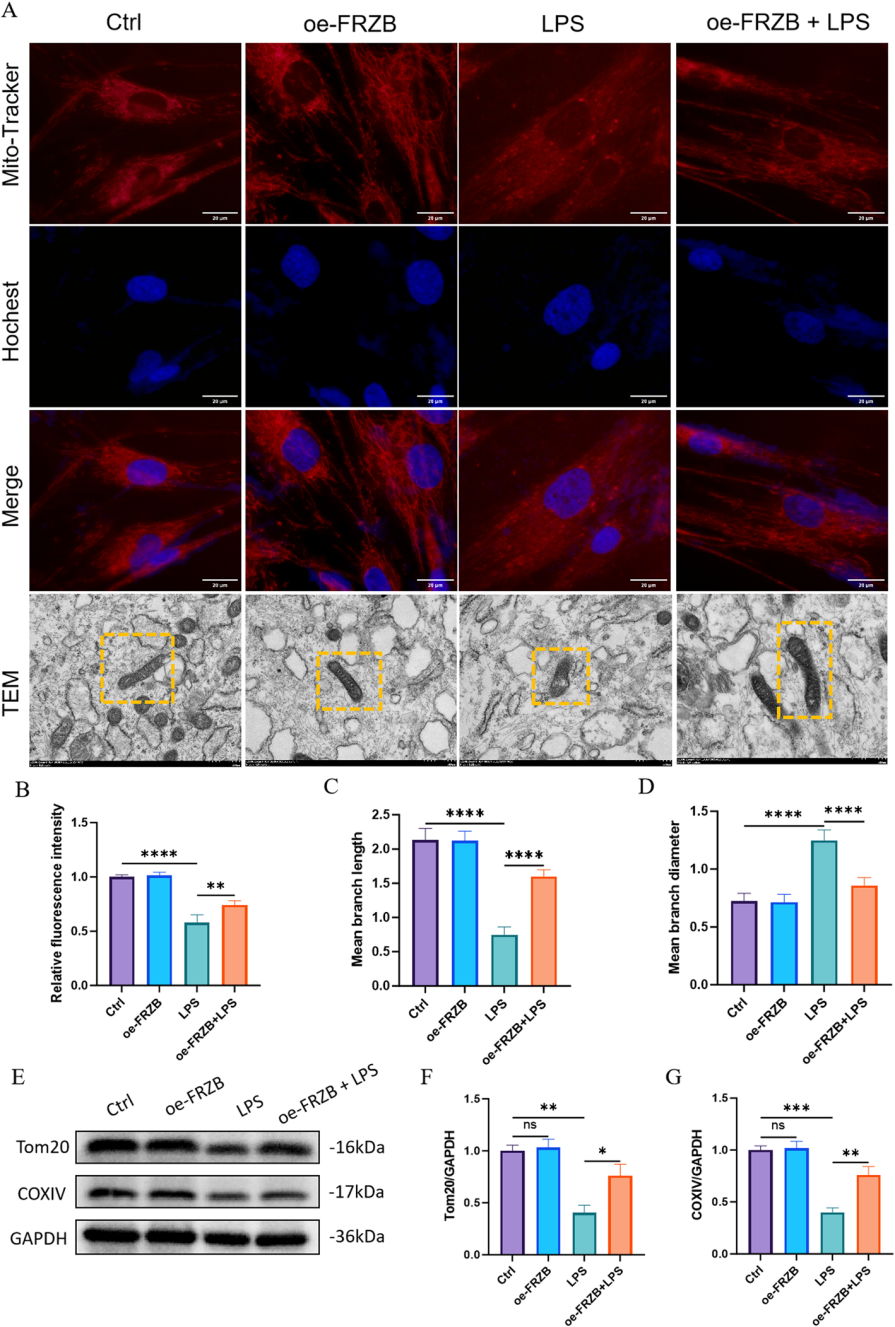
The effects of LPS and FRZB overexpression on mitochondria in PDLSCs. **A** Immunofluorescence co-staining of Mito-Tracker (red, mitochondrial probe) and Hoechst (blue, nuclear stain); scale bar = 20 μm. TEM images (mitochondria in yellow boxes): intact mitochondria in Control/oe-FRZB groups, damaged in LPS group, and partially restored in oe-FRZB + LPS group, scale bar = 100 μm. **B** Relative fluorescence intensity quantification. Control/oe-FRZB groups had higher intensity; LPS/oe-FRZB + LPS groups showed a significant reduction. **C**-**D** Quantification of mitochondrial branch length (**C**) and diameter (**D**). Control/oe-FRZB groups had greater length/diameter; these parameters decreased sharply in LPS group but recovered in oe-FRZB + LPS group. **E** Western blot of Tom20 and COXIV, with GAPDH (36 kDa) as loading Control.** F**-**G** Quantitative analysis: Tom20/GAPDH (**F**) and COXIV/GAPDH (**G**) were reduced in LPS group and restored in oe-FRZB + LPS group (data presented as mean ± SD; One-Way ANOVA; *n* = 3; *P < 0.05, **P < 0.01, ***P < 0.001, ****P < 0.0001)

MitoTracker fluorescence staining showed that mitochondria in the Control and oe-FRZB groups presented a dense reticular structure with strong fluorescent signals. In contrast, the LPS group exhibited weakened fluorescence intensity and disrupted reticular mitochondrial structure, while both the mitochondrial structure and fluorescence intensity were restored in the oe-FRZB + LPS group (Fig. 2A). Transmission electron microscopy (TEM) results further confirmed that LPS-induced mitochondrial morphological abnormalities, including fragmentation, shortened length, swelling, increased diameter, and the breakdown of interconnected tubular mitochondrial networks into isolated small spherical or short rod-like particles, were reversed by FRZB overexpression (Fig. 2A). Quantitative analysis revealed that compared to the control group, the relative fluorescence intensity and mitochondrial branch length were significantly decreased, while the mitochondrial branch diameter was significantly increased in the LPS group. In contrast, these alterations were partially restored in the oe-FRZB + LPS group compared to the LPS group (Fig. 2B-D). Western blot analysis showed no significant difference in the expression of Tom20, an outer mitochondrial membrane protein, between the Control and oe‑FRZB groups. However, Tom20 expression was significantly reduced in both the LPS group and the oe‑FRZB + LPS group, with a more pronounced decrease observed in the latter. This reduction may be attributed to the dissociation of Tom20 into the cytoplasm following mitochondrial damage, where it is subsequently degraded. The expression of COXIV, a mitochondrial respiratory chain protein, was decreased in the LPS group but restored in the oe-FRZB + LPS group, confirming that FRZB overexpression could alleviate LPS-induced damage to mitochondrial respiratory function (Fig. 2E-G).

### LPS causes mitochondrial dysfunctions, and FRZB overexpression can reverse these dysfunctions in PDLSCs

**Fig. 3:**
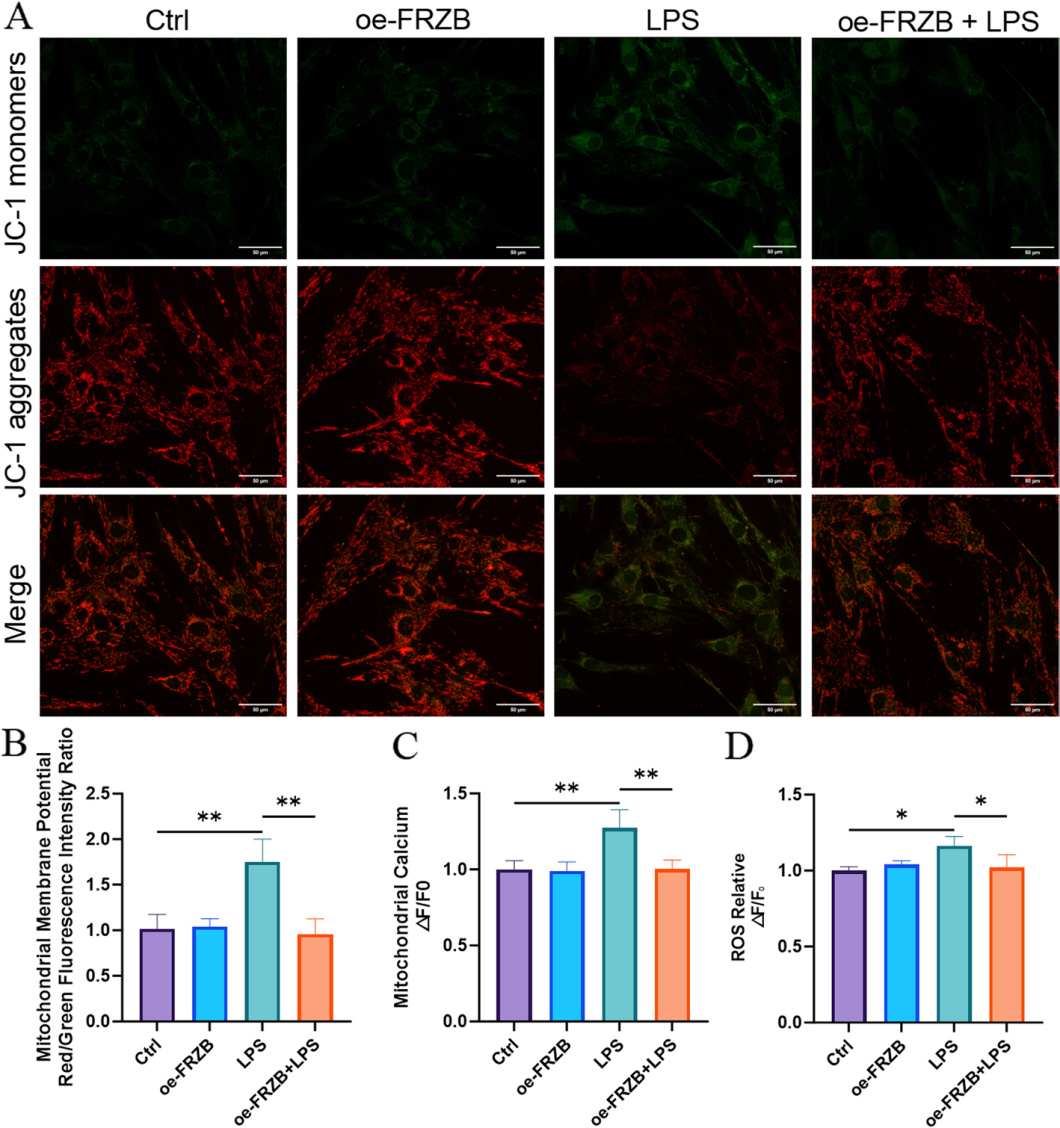
The effects of LPS and FRZB overexpression on mitochondrial membrane potential, ROS, and mitochondrial calcium concentration. **A** Fluorescence microscopy images showing JC-1 monomers, JC-1 aggregates, and merged fluorescence in the Control, oe-FRZB, LPS, and oe-FRZB + LPS groups, Scale bar = 50 μm; **B** Statistical analysis of the red/green fluorescence intensity ratio indicating mitochondrial membrane potential in the Control, oe-FRZB, LPS, and oe-FRZB + LPS groups; **C** Relative fluorescence intensity changes of mitochondrial calcium ions in the Control, oe-FRZB, LPS, and oe-FRZB + LPS groups; **D** Relative fluorescence intensity changes of ROS in the Control, oe-FRZB, LPS, and oe-FRZB + LPS groups (data presented as mean ± SD; One-Way ANOVA; *n* = 3; *P < 0.05, **P < 0.01, ***P < 0.001)

The JC-1 mitochondrial membrane potential assay showed that the Control and oe-FRZB groups displayed intense red and faint green fluorescence. In contrast, the LPS group showed strong green and weak red fluorescence. The oe-FRZB + LPS group exhibited a recovery in red fluorescence and a decrease in green fluorescence compared to the LPS group alone (Fig. 3A). This phenomenon indicates that LPS treatment reduced the mitochondrial membrane potential, and overexpression of FRZB may partially alleviate the decrease in mitochondrial membrane potential caused by LPS. By observing the changes in mitochondrial membrane potential, mitochondrial calcium ion concentration, and ROS levels across different experimental groups, it was found that in the Control group, the mitochondrial membrane potential remained normal, ROS levels were stable, and mitochondrial Ca^2^⁺ concentration was maintained at baseline levels. No significant differences in these parameters were observed between the oe-FRZB group and the Control group. In contrast, the LPS group exhibited a pronounced loss of mitochondrial membrane potential, a significant elevation in mitochondrial Ca^2^⁺, and a substantial increase in ROS levels. Notably, compared to the LPS group, the oe-FRZB + LPS group demonstrated a recovery trend in membrane potential, reduced mitochondrial calcium accumulation, and decreased ROS production, suggesting that FRZB overexpression mitigates LPS-induced mitochondrial dysfunction (Fig. 3B-D).

### LPS inhibits mitophagy, and FRZB overexpression can reverse the inhibition in PDLSCs

**Fig. 4:**
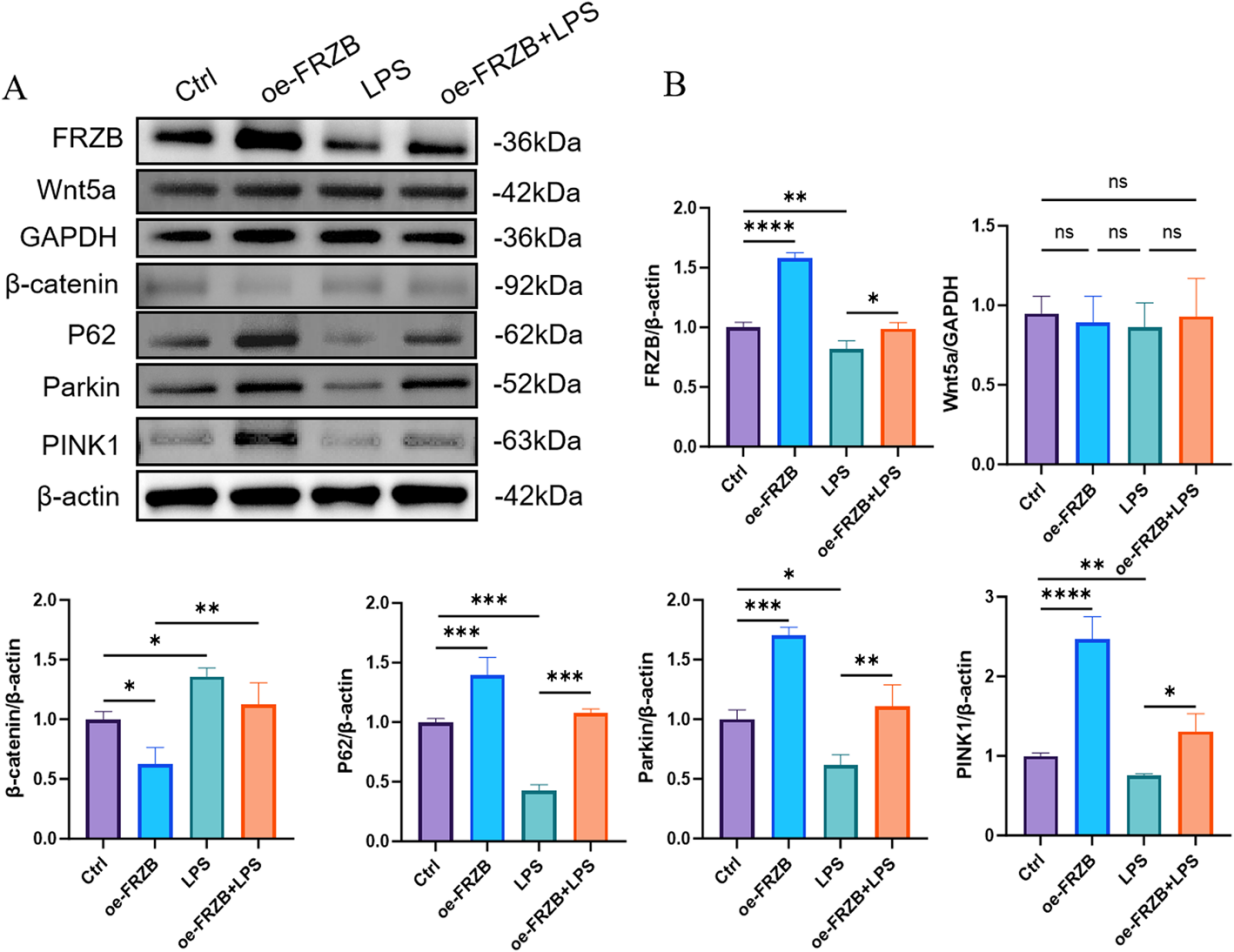
The effects of LPS and FRZB overexpression on the levels of proteins in Wnt pathway and mitophagy. **A** and **B** show that in the LPS group, the expression of FRZB, PINK1, Parkin, and P62 was reduced, while the expression of β-catenin was increased. Conversely, the opposite outcomes were observed in the oe-FRZB group and the oe-FRZB + LPS group (data presented as mean ± SD; One-Way ANOVA; *n* = 3; *P < 0.05, **P < 0.01, ***P < 0.001)

Western blot analysis revealed that LPS stimulation downregulated the expression of mitophagy-related proteins (Pink1 and Parkin) and the autophagy adaptor protein P62, while upregulating β-catenin expression. Conversely, opposite trends were observed in both the oe-FRZB group and the LPS group. These findings indicate that inflammatory conditions activate the Wnt/β-catenin signaling pathways, potentially suppressing autophagy and mitophagy. Overexpression of FRZB appears to attenuate these effects (Fig. 4).

### LPS inhibits autophagy, and FRZB overexpression can reverse the inhibition in PDLSCs

**Fig. 5:**
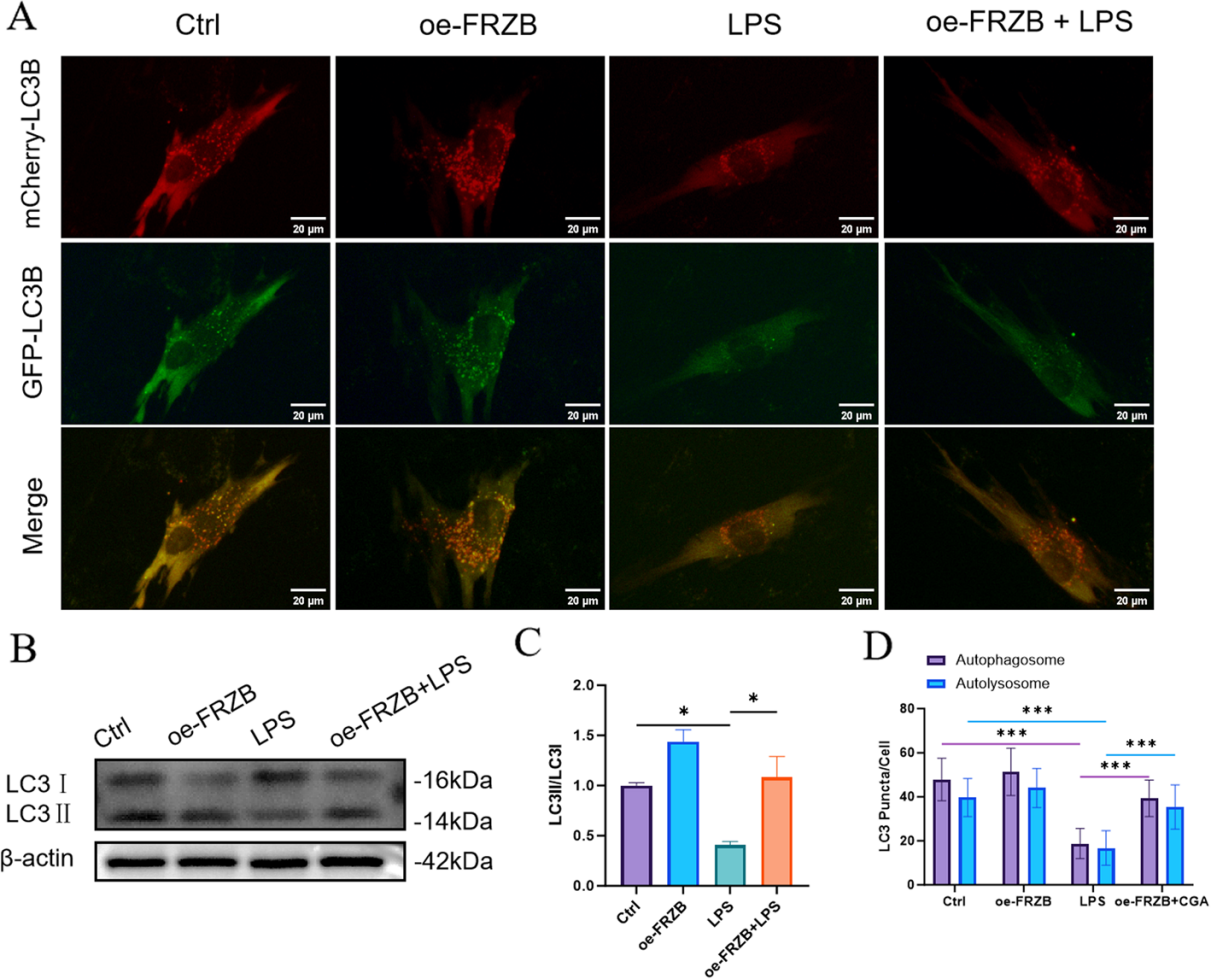
The effects of LPS and FRZB overexpression on autophagy. **A** The Control and oe-FRZB groups displayed more yellow fluorescence, while the LPS group showed less yellow and red fluorescence. The oe-FRZB + LPS group exhibited a recovery in both yellow and red fluorescence compared to the LPS group alone, Scale bar = 20 μm; **B** The expression of LC3 II was lower in the LPS group compared to the Control and oe-FRZB groups. Conversely, the oe-FRZB + LPS group had increased LC3 II expression relative to the LPS group.** C** The LC3 II/I ratio was significantly lower in the LPS group than in the Control and oe-FRZB groups. The oe-FRZB + LPS group had a significantly higher LC3 II/I ratio than the LPS group. **D** The number of autophagosomes and autolysosomes was significantly reduced in the LPS group compared to the Control and oe-FRZB groups. The oe-FRZB + LPS group had a notable increase in the number of autophagosomes and autolysosomes compared to the LPS group (data presented as mean ± SD; One-Way ANOVA; *n* = 3; *P < 0.05, **P < 0.01, ***P < 0.001)

mCherry-GFP-LC3 dual fluorescence staining and western blot analysis revealed: In the Control group, abundant yellow fluorescence indicated a certain number of autophagosomes. The oe-FRZB group exhibited a similar distribution and quantity of yellow puncta compared to the Control, suggesting that FRZB overexpression did not significantly affect basal autophagic activity. In the LPS group, a marked reduction in yellow puncta was observed, accompanied by a decrease in red fluorescence and downregulation of the LC3II/LC3I ratio, indicating that LPS may suppress autophagic flux or disrupt autophagosome maturation and degradation, leading to reduced autophagosome accumulation. In the oe-FRZB + LPS group, yellow puncta were notably restored, red fluorescence was partially intensified, and the LC3II/LC3I ratio was higher than that in the LPS group (Fig. 5A-C). Furthermore, both autophagosomes and autolysosomes were significantly fewer in the LPS group compared to the Control and oe-FRZB groups. In contrast, the oe-FRZB + LPS group showed a significant increase in autophagosomes and autolysosomes relative to the LPS group (Fig. 5D). Collectively, these results suggest that FRZB overexpression may restore autophagic activity and promote autophagosome formation and/or maturation under conditions of LPS-induced autophagy impairment.

### LPS reduces the osteogenic capacity of PDLSCs, and FRZB overexpression can reverse this impact

**Fig. 6:**
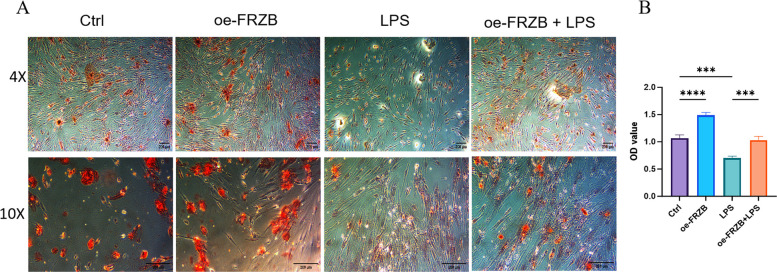
The effects of LPS and FRZB overexpression on osteogenic capacity of PDLSCs. **A** The Control group displayed large, dark red nodules. The LPS group had smaller nodules with lighter coloration compared to the Control group. The oe-FRZB group was similar to the Control group, with a darker color. The LPS + oe-FRZB group showed an increase in the number of red nodules and a darker color compared to the LPS group, Scale bar = 200 μm;** B** The OD values of the LPS group were significantly lower than those of the Control and oe-FRZB groups. The OD values of the oe-FRZB + LPS group were markedly higher than those of the LPS group (data presented as mean ± SD; One-Way ANOVA; *n* = 3; *P < 0.05, **P < 0.01, ***P < 0.001,****P < 0.0001)

Following 21 days of osteogenic induction, PDLSCs in the Control group exhibited prominent dark red nodules. In contrast, the LPS group displayed smaller and less intensely stained nodules. The oe-FRZB group resembled the Control group, with similarly dark red nodules. Notably, the LPS + oe-FRZB group showed increased nodule formation and deeper red coloration compared to the LPS group (Fig. 6A). Quantitative analysis revealed that the optical density (OD562) values in the LPS group were significantly lower than those in both the Control and oe-FRZB groups. Conversely, the LPS + oe-FRZB group exhibited markedly higher OD values than the LPS group (Fig. 6B). These results indicate that LPS-induced periodontitis conditions inhibit the osteogenic potential of PDLSCs, whereas FRZB overexpression effectively rescues osteogenic differentiation under inflammatory challenge.

## Discussion

Alveolar bone resorption in periodontitis remains a major therapeutic challenge owing to its multifactorial pathogenesis (Huang et al. 2020). Under inflammatory microenvironments, the osteogenic differentiation capacity of PDLSCs is markedly impaired (Wu et al. 2024). Given their accessibility, high proliferative potential, and multipotency, PDLSCs represent a promising candidate for regenerative applications (Parichehr et al. 2023). In particular, their osteogenic potential is paramount for the regeneration of bone defects associated with periodontitis (Arora et al. 2022).

Surface molecular marker analysis of PDLSCs (CD45⁻/CD90⁺/CD146⁺) confirmed their mesenchymal origin and stem-like properties. CCK-8 assays revealed that the inflammatory microenvironment significantly suppressed both proliferation and viability of PDLSCs. In Alizarin Red staining assays, the LPS group exhibited mineralized nodules with reduced size and staining intensity compared to the Control, indicating impaired osteogenic differentiation—consistent with clinical observations of alveolar bone loss and defective osteogenesis in periodontitis patients (Tsuchida and Nakayama 2023). In contrast, the LPS + oe-FRZB group showed increased formation of intensely stained mineralized nodules relative to the LPS group. These results demonstrate that FRZB overexpression rescues the osteogenic potential of PDLSCs under inflammatory conditions.

In this study, the interaction between FRZB and Wnt5a was confirmed by co-immunoprecipitation assays. Molecular docking further elucidated the specific amino acid residues mediating their binding. Certain Frizzled receptors, including FZD4, FZD5, and FZD7, are known to bind Wnt5a and activate both canonical and non-canonical Wnt signaling pathways (Hiong et al. 2015). Notably, FRZB contains a cysteine-rich domain (CRD) structurally similar to those in Frizzled receptors. Via this domain, FRZB binds extracellular Wnt ligands and competitively inhibits their interaction with Frizzled receptors, thereby suppressing downstream signaling (Li et al. 2024). In the present work, LPS treatment resulted in a significant increase in β-catenin expression, accompanied by a decrease in FRZB expression. These findings suggest that the inflammatory microenvironment might downregulate FRZB expression, activating the Wnt/β-catenin signaling pathway and consequently impairing the osteogenic differentiation potential of PDLSCs.

Furthermore, PDLSCs treated with LPS displayed disrupted mitochondrial ultrastructure, accompanied by elevated mitochondrial membrane potential, increased accumulation of mitochondrial calcium, and enhanced ROS production. In contrast, overexpression of FRZB effectively attenuated these pathological changes. Collectively, these findings suggest that the inflammatory microenvironment compromises mitochondrial function in PDLSCs. Mitophagy, as a selective form of autophagy, contributes to the clearance of damaged mitochondria, thereby conferring protection against cellular apoptosis (Liu et al. 2012). In this study, LPS treatment resulted in the downregulation of PINK1, Parkin, and P62, along with a decreased LC3-II/LC3-I ratio. These findings indicate that dysregulated autophagy may impair the osteogenic differentiation potential of PDLSCs. The proposed mechanism involves LPS-induced suppression of mitophagy, compromising the cellular clearance of damaged mitochondria. Consequently, these mitochondria accumulate and release ROS and pro-apoptotic factors, promoting cell death or apoptosis, as reflected by reduced PINK1 and Parkin expression. Furthermore, LPS appears to disrupt the autophagic flux, evidenced by enhanced degradation of LC3-II and impaired conversion of LC3-I to LC3-II, leading to reduced LC3 expression, fewer autophagosomes, and overall suppression of autophagy. This autophagic dysfunction may further exacerbate intracellular oxidative stress, thereby attenuating the osteogenic capacity of PDLSCs. Collectively, these results suggest that the inflammatory microenvironment inhibits osteogenic differentiation in PDLSCs by inducing mitochondrial dysfunction. This observation aligns with previous reports indicating that mitochondrial impairment in PDLSCs within the inflammatory milieu of periodontitis contributes to diminished osteogenic potential (Li et al. 2022).

**Fig. 7:**
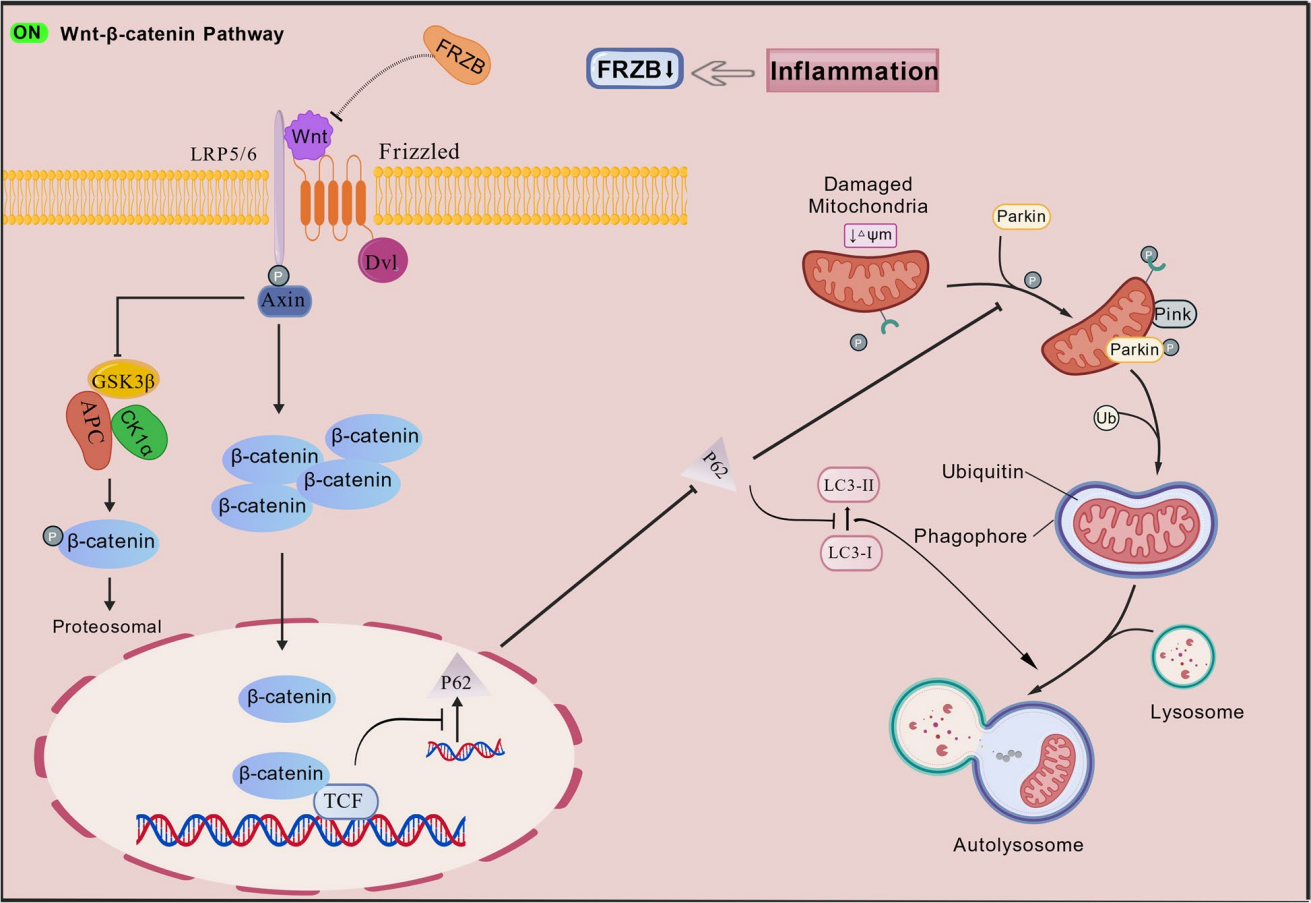
Schematic illustration of the mechanism by which FRZB modulates mitochondrial function via the Wnt/β-catenin signaling pathway (Jiang et al. 2025)

Notably, this study confirms that overexpression of the FRZB gene partially restores mitochondrial function under inflammatory conditions. The mechanism involves the binding of Wnt5a to frizzled receptors on the cell membrane during periodontitis, which concurrently activates the Wnt/β‑catenin signaling pathway. This activation promotes the nuclear accumulation of β‑catenin, thereby inhibiting the transcription of the P62/SQSTM1 gene through transcription factors such as TCF4, directly reducing p62 mRNA levels and de novo protein synthesis. Meanwhile, under inflammatory conditions, the autophagy process is suppressed, and the degradation of p62 via the autophagy-lysosomal pathway is hindered, which would theoretically lead to p62 accumulation. However, within the specific experimental system and treatment timeframe of this study, the transcriptional repression of p62 mediated by TCF4 upon Wnt/β‑catenin signaling activation outweighs the effect of reduced p62 degradation due to inhibited autophagy, ultimately resulting in a decrease in total p62 protein levels. When P62 transcription is reduced, even if the upstream PINK1/Parkin pathway is activated and has labeled damaged mitochondria, the lack of p62 as a mediator prevents these labeled mitochondria from being effectively encapsulated into autophagosomes for degradation. This directly leads to the accumulation of damaged mitochondria within the cell, resulting in mitochondrial dysfunction. (Petherick et al. 2013) (Fig. 7).

When Wnt proteins bind to FZD receptors and LRP5/6 co-receptors, the cytoplasmic region of LRP5/6 is phosphorylated by GSK3β. This leads to the recruitment of Dvl and axin proteins to the cytoplasmic domains of the Frizzled and LRP5/6 receptors, respectively. Activated Dvl disrupts the destruction complex and inhibits GSK3β-mediated phosphorylation of β-catenin, thereby preventing its degradation. Consequently, β-catenin accumulates in the cytoplasm, translocates into the nucleus, and binds to TCF transcription factors, which suppresses the transcription and expression of P62. The downregulation of P62 reduces the stability and activation of PINK1, impairs the recruitment and activation of Parkin, blocks the mitophagy pathway, and ultimately leads to mitochondrial dysfunction. In contrast, FRZB can inhibit Wnt signaling by simultaneously binding to both Wnt proteins and Frizzled receptors. Under inflammatory conditions, downregulation of FRZB activates the Wnt/β-catenin pathway, contributing to mitochondrial dysfunction.

Collectively, this study elucidates a potential mechanism by which the inflammatory microenvironment regulates PDLSC osteogenesis, providing novel insights into periodontitis pathogenesis. Our findings indicate that FRZB modulates mitochondrial functions, including ROS generation, apoptosis, and mitophagy, through regulation of Wnt signaling pathway, thereby determining the osteogenic capacity of PDLSCs under inflammation. However, limitations warrant consideration: 1) While this study provides key insights through in vitro experiments, the currently adopted LPS in vitro model cannot fully replicate the complex microenvironment of periodontitis in vivo. Future validation via animal experiments and other approaches is still required to establish the pathophysiological relevance of these findings; 2) Although the FRZB-Wnt5a-mitochondrial axis represents a key regulatory pathway, the mechanisms governing osteogenic differentiation in inflammatory microenvironments likely involve intricate multi-pathway and multi-factor interplay. Thus, a comprehensive dissection of this complex regulatory network will require integrated approaches across multiple experimental models.

## Conclusions

In this experiment, the establishment of an inflammatory microenvironment model in vitro revealed a significant suppression of osteogenic differentiation capacity in PDLSCs. This inhibition appears to be mediated by the downregulation of FRZB under inflammatory conditions, which activates the Wnt/β-catenin signaling pathway, leading to mitochondrial dysfunction. Collectively, these results indicate that the inflammatory microenvironment modulates osteogenic differentiation of PDLSCs via the FRZB-Wnt5a-mitochondrial axis, providing novel mechanistic insight for the development of therapeutic strategies against periodontitis.

## Materials and methods

### Cell culture

PDLSCs were isolated from impacted third molars extracted from healthy donors (aged 18–30 years). All procedures were approved by the Ethics Committee of the Affiliated Stomatology Hospital of Nanchang University (Permit No. 2023011), and written informed consent was obtained from all donors. Exclusion criteria comprised severe caries, periapical or periodontal pathologies, history of smoking, and use of certain medications. Immediately following extraction, teeth were immersed in phosphate-buffered saline (PBS, Solarbio, P1020, China) containing antibiotics. Periodontal ligament tissues were gently scraped from the root surface, minced into 1–2 mm^3^ fragments, and evenly plated in culture dishes. The tissue fragments were allowed to adhere to the dish surface for 2–4 h, after which complete growth medium (α-MEM (BDBIO, L105-500, China) supplemented with 20% fetal bovine serum (FBS, ExCell Bio, FBS500, Brazil) and 1% Penicillin–Streptomycin Liquid (Solarbio, Cat: P1400, China) was carefully added. The cultures were maintained in a humidified incubator at 37 °C with 5% CO₂, and the medium was changed every 2–3 days until cells migrated out from the tissue blocks and reached confluence. Passage 3 (P3) PDLSCs were used for identification. For flow cytometry, cells (1 × 10^5^/mL) were seeded in 6-well plates, harvested at 70–80% confluency, and incubated with fluorochrome-conjugated antibodies against FITC-CD45 (Elabscience, E-AB-F1417D-20, China)、FITC-CD146 (Elabscience, E-AB-F1167C-20, China)、PE-CD90 (Elabscience, E-AB-F1137C-20, China) for 30 min at 4 °C. After PBS washing, surface marker expression was analyzed by a full-spectrum multi-color flow cytometer (Sony, ID700, Japan). Data analysis was performed using FlowJo software (version 10).

### Inflammatory cell model establishment

To establish the inflammatory cell model, PDLSCs were cultured to 80% confluency and treated with different concentrations (0, 2, 5, 10 μg/mL) of lipopolysaccharide (LPS, Solarbio, Cat: L8880, China) to induce a pro-inflammatory response.

Protein levels of inflammatory factors (IL-6, IL-1β, TNF-α) were determined by western blot analysis. Cell viability was assessed using the Cell Counting Kit-8 (CCK-8, Beyotime, C0038, China) assay, in which cells were incubated with the CCK-8 reagent followed by measurement of the absorbance at 450 nm (optical density, OD). Differences in OD values among experimental groups indicated the effects of LPS treatment on cellular activity.

### Alizarin Red staining

PDLSCs were cultured in osteogenic induction medium OIM (α-MEM, 10% FBS, 10 mM β-glycerophosphate (Beyotime, ST637, China), 50 μg/mL ascorbic acid (Solarbio, Cat A8100, China), and 100 nM dexamethasone (Beyotime, ST1254, China) for 21 days with medium changes every 3 days. Experimental groups included: 1) α-MEM group: cultured using only α-MEM basal medium without the addition of osteogenic induction factors. 2) Control group: cultured solely in OIM. 3) LPS group: cultured in OIM supplemented with 10 μg/mL LPS. 4) Overexpressing FRZB (OBiO Technology, H45358, China) group (oe-FRZB group): PDLSCs were transfected with lentiviral vectors carrying the FRZB overexpression plasmid and cultured in OIM. 5) Overexpressing FRZB + LPS group (oe-FRZB + LPS group): PDLSCs were transfected with lentiviral vectors carrying the FRZB overexpression plasmid and cultured in OIM supplemented with 10 μg/mL LPS.

After 21 days, cells were fixed with 4% paraformaldehyde for 15 min, washed with PBS, and stained with 1% alizarin red solution (pH 4.2) for 10 min. Mineralized nodules were photographed following PBS washes. Calcium nodules were dissolved using 10% cetylpyridinium chloride. After complete dissolution, they were loaded into 96-well plates, and the OD value at a wavelength of 562 nm was measured.

### Mitochondrial function analysis

Cells from each group were stained with mitochondrial membrane potential detection kit (JC-1, Beyotime, C2003S, China), reactive oxygen species fluorescent dye (DCFH-DA, Beyotime, S0034S, China), and mitochondrial calcium ion detection kit (Rhod-2 AM, Beyotime, S1062S, China) at 37℃, 5% CO₂ for 30 min in the dark. After staining, the cells were observed and photographed under a fluorescence microscope. Additionally, the fluorescence intensity of each group of cell suspension was measured using a multifunctional microplate reader (Moleculardevices, SpectraMax iD5, USA) to quantitatively analyze changes in mitochondrial membrane potential, ROS levels, and mitochondrial calcium ion concentration.

### Mito-Tracker Staining and mCherry-GFP-LC3 Fluorescent Staining

After 24-h treatments, cells were washed with serum-free medium and incubated with 1 μM Mito-Tracker Red CMXRos (Beyotime, C1035, China) (37 °C, 30 min, protected from light). Following two washes, nuclei were counterstained with 1 μg/mL Hoechst 33,342 (Beyotime, C1022, China) (5 min, dark). After three additional washes, cells were mounted with antifade reagent and coverslipped for imaging using a laser scanning confocal microscope (Zeiss, LSM 980, Germany).

Cells were infected with 5 MOI Ad-mCherry-GFP-LC3B (Beyotime, C3011, China) adenovirus for 48 h to express fluorescent-tagged proteins. The medium was aspirated, and cells were washed twice with serum-free medium. The cell samples were sealed using the sealing agent. Fluorescent puncta (indicating autophagosomes) were visualized by a laser scanning confocal microscope.

### Transmission electron microscope

Cells from each group were centrifuged (≤ 1500 rpm, 5 min) to form a pellet approximately the size of a mung bean, which was then resuspended in electron microscopy fixative (Servicebio, G1102-100ML, China). The samples were dehydrated and embedded using epoxy resin 812 (Servicebio, GP2001, China) as the embedding medium. The embedded specimens were then sectioned into ultrathin slices with a thickness of 60–80 nm. The stained sections were mounted on copper grids for electron microscopy to complete sample preparation. The ultrastructural morphology of mitochondria was observed and analyzed using transmission electron microscopy (TEM) (Hitachi, HT7800, Japan).

### Western blot

PDLSCs were lysed in RIPA buffer (ThermoFisher, 89901, USA), and protein concentrations were quantified using a BCA protein assay kit (Takara, T9300A, USA). Proteins were separated by 10% SDS-PAGE and transferred onto polyvinylidene fluoride (PVDF, Sigma, GVWP04700, Germany) membranes. After blocking with 5% skim milk for 1 h at room temperature, membranes were incubated overnight at 4 °C with primary antibodies against the following targets: Wnt5a (Proteintech, 55,184–1-AP, China), β-catenin (Abmart, PK02151, China), PINK1 (Proteintech, 23,274–1-AP, China), Parkin (Proteintech, 14,060–1-AP, China), LC3B (Proteintech, 14,600–1-AP, China), and P62 (Abmart, PQA1642, China). The next day, membranes were washed with TBST and incubated with HRP-conjugated Goat Anti-Rabbit IgG (H + L)/Anti-Mouse IgG (H + L) (Proteintech, SA00001-2/SA00001-1, China) for 1 h at room temperature. Protein bands were visualized using enhanced chemiluminescence (ECL, UElandy S6009L, China) reagent and imaged with a chemiluminescence detection system (Servicebio, SCG-W3000 PLUS, China). Band intensities were quantified densitometrically to evaluate protein expression levels.

### Co-immunoprecipitation (Co-IP) assay

An appropriate amount of PDLSCs lysate was respectively incubated with Wnt5a and FRZB antibodies overnight at 4 °C. Protein A/G magnetic beads (MedChemExpress, HY-K0202, China) were then added, and the mixture was incubated for 2 h. The beads were subsequently separated using a magnetic rack and washed. Afterward, 2 × SDS-PAGE loading buffer was added, and the immunocomplexes were dissociated by boiling for 5 min. The interaction between FRZB and Wnt5a was observed by western blot analysis. The binding sites of the Wnt5a and FRZB protein macromolecules were simulated through molecular docking technology.

### Statistical analysis

All experiments were conducted at least in triplicate, and the results are presented as the mean ± standard deviation (SD). The two-tailed unpaired Student's t-test wasused for two-group comparisons. One-way analysis of variance (ANOVA) was applied for the comparison of three or more groups. All statistical analyses were carried out by GraphPad Prism version 9 (GraphPad Software Inc., USA). The threshold of statistical significance was set as *p* < 0.05.

## Supplementary Information

Supplementary Material 1. Supplementary Figures. Fig. S1. Culture of PDLSCs and estabishment of periodontitis cell model. Fig. S2. 2. LPS-treated PDLSCs exhibit osteogenic inhibition.

## Data Availability

The data that support the findings of this study are available from the corresponding author upon reasonable request.

## Abbreviations

PDLSCs: Periodontal Ligament Stem Cells
LPS: Lipopolysaccharide
ROS: Reactive Oxygen Species
Wnt5a: Wingless-Type MMTV Integration Site Family, Member 5A
FRZB: Frizzled-Related Protein
ATP: Adenosine Triphosphate
OIM: Osteogenic Induction Medium
α-MEM: Minimum Essential Medium Alpha
FBS: Fetal Bovine Serum
CCK-8: Cell Counting Kit-8
OD: Optical Density
PBS: Phosphate-Buffered Saline
SDS-PAGE: Sodium Dodecyl Sulfate–Polyacrylamide Gel Electrophoresis
PVDF: Polyvinylidene Fluoride
ECL: Enhanced Chemiluminescence
Co-IP: Co-Immunoprecipitation
JC-1: 5,5',6,6'-Tetrachloro-1,1',3,3'-tetraethylbenzimidazolylcarbocyanine iodide
DCFH-DA: 2',7'-Dichlorodihydrofluorescein Diacetate
Rhod-2 AM: Rhodamine-2 Acetoxymethyl Ester
MOI: Multiplicity of Infection
LC3I/II: Microtubule-associated protein 1 light chain 3-I/II
P62: Sequestosome 1
PINK1: PTEN-Induced Kinase 1
GAPDH: Glyceraldehyde-3-Phosphate Dehydrogenase
β-actin: Beta-Actin
TNF-α: Tumor Necrosis Factor-Alpha
IL-1β: Interleukin-1 Beta
IL-6: Interleukin-6
BCA: Bicinchoninic Acid
TEM: Transmission Electron Microscopy
ANOVA: Analysis of Variance
