# Supplementary material for: FRZB regulates the osteogenic differentiation of periodontal ligament stem cells in an inflammatory microenvironment through Wnt5a-mitochondrial axis
Source: Cell Regen. 2026 Feb 6;15:9. doi: 10.1186/s13619-026-00283-z (PMC12881248; doi:10.1186/s13619-026-00283-z)
Supplement: Supplementary file 1 — Supplementary Material 1. Supplementary Figures. Fig. S1. Culture of PDLSCs and estabishment of periodontitis cell model. Fig. S2. 2. LPS-treated PDLSCs exhibit osteogenic inhibition. [file 13619_2026_283_MOESM1_ESM.docx]

**Supplementary Information**

1. **Culture of PDLSCs and estabishment of periodontitis cell model**

**
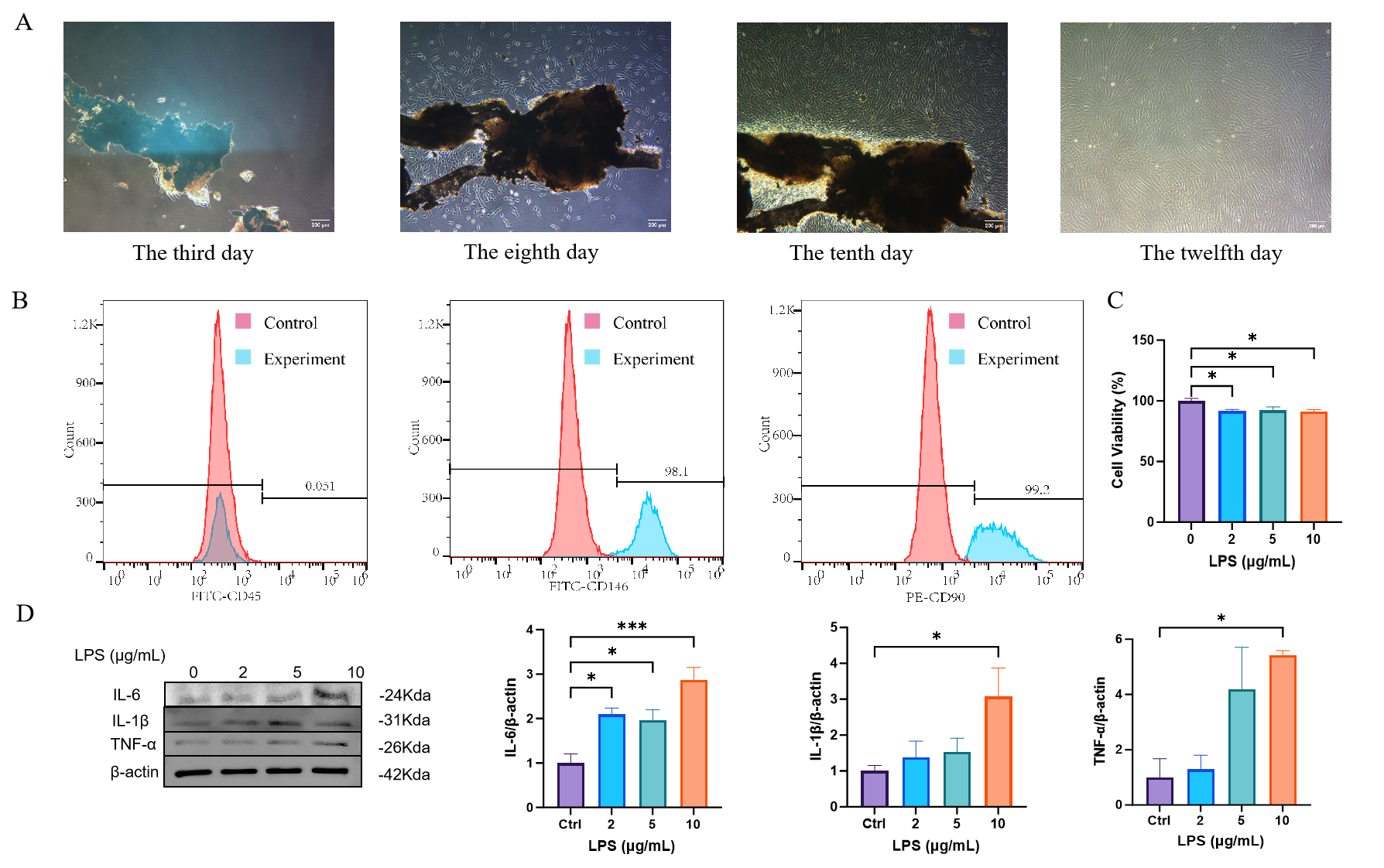
**

**Fig S1. Culture of PDLSCs and establishment of the inflammatory cell model.**

1. **.**Primary PDLSCs exhibited a uniform bipolar spindle-shaped morphology with fusiform centers under optical microscopy, growing in characteristic whorl-like patterns with minimal size variation, Scale bar = 50 μm; **(B).** Flow cytometry confirmed their mesenchymal stem cell identity, showing ≥95% positivity for CD90 and CD146, and negative for CD45 expression (≤2%). **(C).**CCK-8 assays showed that LPS treatment significantly reduced cell viability. **(D).** The expression levels of inflammatory cytokines (IL-1β, TNF-α, IL-6) showed LPS dose-dependent upregulation, peaking at 10 μg/mL. (data presented as mean ± SD; One-Way ANOVA; n = 3; *P < 0.05, **P < 0.01, ***P < 0.001) .
2. **LPS-treated PDLSCs exhibit osteogenic inhibition**

**
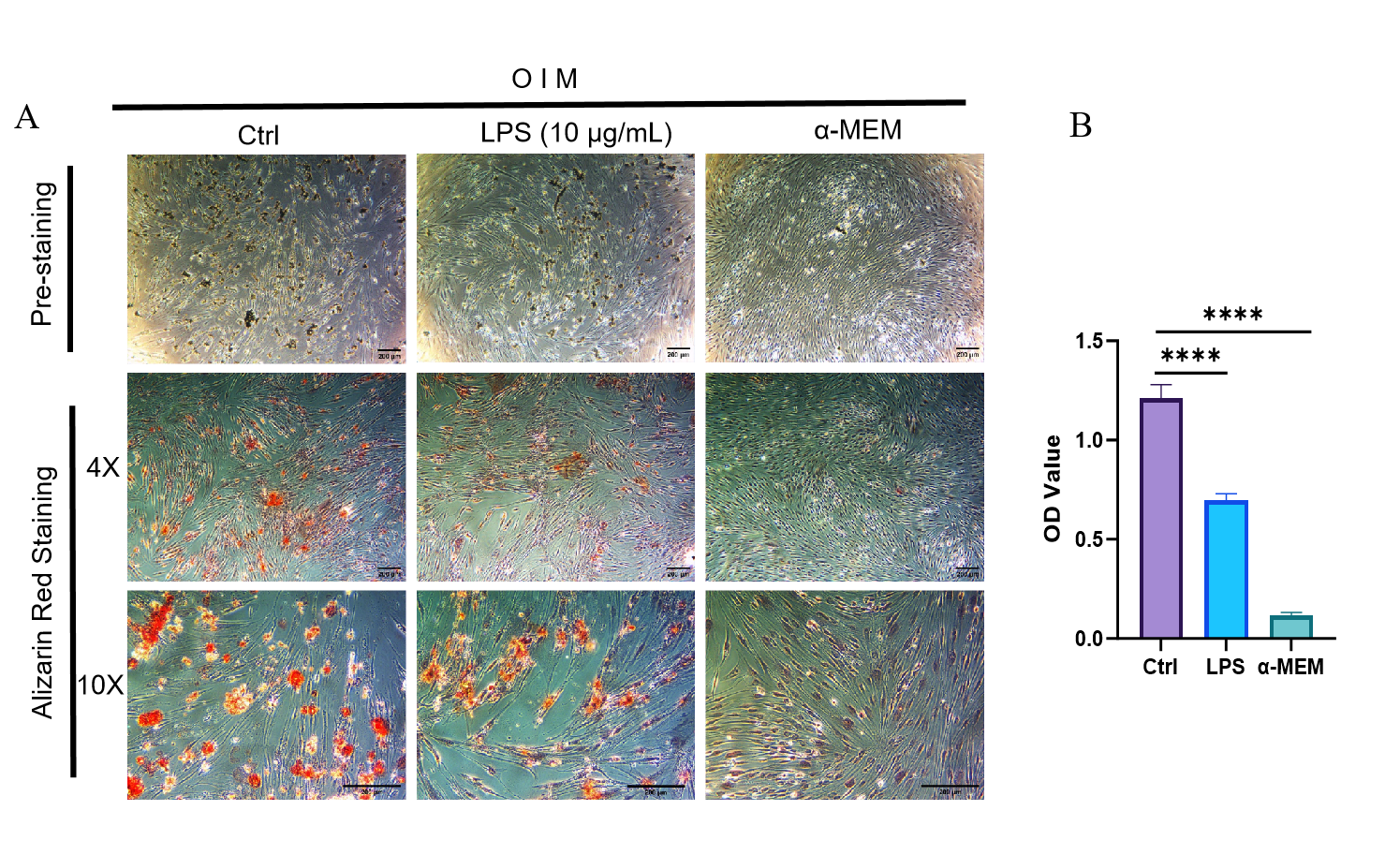
**

**Fig S2. The effect of LPS on osteogenesis in PDLSCs was detected by Alizarin Red staining.**

**(A).** Alizarin red staining revealed the Control group exhibited extensive dark-red mineralized nodules. In contrast, the LPS group showed significantly reduced nodule formation with lighter coloration (The red areas represent mineralized regions, and 4× and 10× indicate the magnification factors)，Scale bar = 200 μm; **(B).** The OD value of the LPS group was significantly lower than that of the Control group (data presented as mean ± SD; One-Way ANOVA; n = 3; **** P < 0.0001).
